# Perspectives and attitudes of South African medical professionals towards active euthanasia

**DOI:** 10.4102/safp.v66i1.5926

**Published:** 2024-09-27

**Authors:** Willem-Johan Steyn, Mukinay C. Bondo

**Affiliations:** 1 Department of Family Medicine, Faculty of Health Sciences, University of Pretoria, Pretoria, South Africa

**Keywords:** euthanasia, active euthanasia, medical professionals, qualitative, thematic analysis

## Abstract

**Background:**

Active euthanasia is a controversial subject both globally and in South Africa. Recent legal cases have kept this topic in the public discourse. Yet, there remains a dearth of quality local research on this issue. This study aimed to explore the views of medical professionals towards active euthanasia to gain a better understanding of this phenomenon in South Africa.

**Methods:**

A descriptive-interpretive qualitative research design, using semi-structured in-depth interviews of purposively sampled South African medical professionals, was used to collect the data. The data were analysed using an inductive thematic analysis framework, which included familiarisation, coding, summarising, theme development and thematic review, revision and interpretation.

**Results:**

Four general themes, with sub-themes, were identified and inductively analysed: modern medical practice and euthanasia; the problem of suffering in end-of-life care; euthanasia is complex; palliative care in South Africa is poor. Some themes and sub-themes were common to all participants while other themes underscored more diverse views – often because of religious factors.

**Conclusion:**

Active euthanasia is a complex and nuanced issue. An understanding of the perceptions and attitudes of medical professionals will contribute to the overall discussion of this phenomenon in ethical, legal, social and political spheres in South Africa. This is of utmost importance given the relevance of this topic in South Africa in the 21st century.

**Contribution:**

This study highlights the complex nature of active euthanasia in South Africa among medical professionals while providing a greater understanding of its nuances and the strengths and weaknesses of arguments for or against it.

## Introduction

Active euthanasia – voluntary euthanasia (VE) and physician-assisted suicide (PAS) – has been a controversial subject for a considerable period. This is not only true globally but also in South Africa.^1^ Although practiced in ancient times, this topic has reached pertinence in the last few decades with the increasing emphasis placed on respecting individual autonomy, especially in the developed world, as well as the rapid advances in medical science.^2^ With the ability to prolong life almost indefinitely with the aid of artificial means, the issue of euthanasia and PAS is a discussion that cannot be ignored.^3^ This is especially true in South Africa given the number of high-profile legal cases concluded and those still ongoing.^4^

Voluntary euthanasia is defined as the ending of a person’s life, at their own voluntary and competent request, by a medical professional through the ingestion of drugs.^5^ This is in contrast to nonvoluntary or involuntary euthanasia that are usually equated with murder.^6^ Physician-assisted suicide, by comparison, is defined as a medical professional intentionally helping a person to end their life by providing drugs for self-administration at that person’s voluntary and competent request.^5^ These two concepts (VE and PAS) are often termed active euthanasia to differentiate it from passive euthanasia (withholding and withdrawing of treatment).

As mentioned, despite the number of high-profile legal cases that have featured prominently in the media, there is a paucity of research surrounding this concept in South Africa. This is particularly true with regard to the views of medical professionals, who would play a central role in active euthanasia if it were ever legalised in South Africa. Currently, all forms of active euthanasia (VE and PAS) are illegal despite draft legislation already being proposed in the late 1990s.^7^

Locally, except for quantitative research conducted by Landman at a single large academic health institution and one by Brits et al. on private medical practitioners, there is scant data on the views of qualified medical professionals surrounding the euthanasia debate.^8,9^ In terms of qualitative research, except for a mixed quantitative/qualitative study conducted on medical students in South Africa, there are no published qualitative studies on the perspectives of medical professionals towards active euthanasia currently.^10^

A critical appraisal of the literature reveals that religion, and more specifically religiosity, is the strongest factor accounting for people’s differing attitudes towards active euthanasia. Burdette et al. consistently found that conservative religious groups in the United States were more opposed to active euthanasia than more liberal religious groups or non-religious people.^11^ Religiosity was also a strongly correlated factor as high religiosity accounted for more negative attitudes towards active euthanasia. Another consistent finding emanating from the global literature has been that medical professionals are more opposed to active euthanasia than the general public.^12,13^ The argument advanced most often for this finding has been that the practice conflicts with traditional medical ethics and the role of the physician as a healer.^14^ In addition, it has been consistently found that those medical professionals more involved in end-of-life care (such as geriatricians, oncologists, palliative care specialists) oppose active euthanasia more than their counterparts in other medical specialities.^15,16^

Given the strong and ambivalent views of medical professionals towards active euthanasia, the authors believed that a descriptive-interpretive qualitative study, using in-depth interviews, would shed greater light on this complex notion: by asking the how and why instead of the how many or how much of quantitative research.^17^ The aim of the study was to broadly explore the perspectives and attitudes of South African medical professionals towards active euthanasia.

## Research methods and design

### Study design

A descriptive-interpretive qualitative research design was chosen as it allows investigation of a concept through in-depth exploration of individuals’ views, experiences and feelings.

### Setting

Medical professionals, in any medical discipline, practicing within South Africa in either public or private practice identified by the researchers. There was no specific setting in which this study was conducted as any medical professional practicing within South Africa, and who satisfied the inclusion criteria, could participate.

### Participant selection and sampling strategy

Purposive sampling was used to identify medical professionals practicing within South Africa. The researchers purposively chose participants from across the country with a diverse range of demographic characteristics to reflect South Africa’s multicultural identity. Participants were either known in some capacity by the researchers or were suggested by others. The researchers did not choose any participant in whom they had previously discussed the subject matter and did not discuss it prior to conducting the interview. The Health Professions Council of South Africa (HPCSA) database was used to ensure that candidates were suitable for participation as per the inclusion and exclusion criteria.^18^ They were subsequently invited to take part in an in-depth interview. The inclusion and exclusion criteria were:

Inclusion criteria: Any qualified, HPCSA registered (MP number) and practicing medical professionals within South Africa (medical professionals denotes doctors with a medical degree – as opposed to other healthcare professionals).

Exclusion criteria: Medical professionals not registered with the HPCSA. Medical professionals who are registered with the HPCSA but do not have an MP number. Medical professionals who have an MP number but are registered as community service medical practitioners.

Medical interns and community service medical practitioners were excluded because of these professionals having limited practical experience and not being confined for any reasonable length of time in a specific discipline. The researchers did not delineate between qualified specialists or medical officers/registrars/general practitioners working within a specialist field as the researchers do not believe that a post-graduate qualification necessarily makes a medical professional experience within a specific field more valuable.

### Data collection

The interviews were scheduled at the participants’ convenience and were conducted either in person (face-to-face) or online over a 12-month period in 2022. Interviews were conducted only once the participant had read the informed consent form and signed it. The interviews were conducted one on one by the lead researcher to protect anonymity. Only the lead researcher knew the identity of each participant, and only this researcher had and still has access to the signed informed consent forms.

**FIGURE 1:**
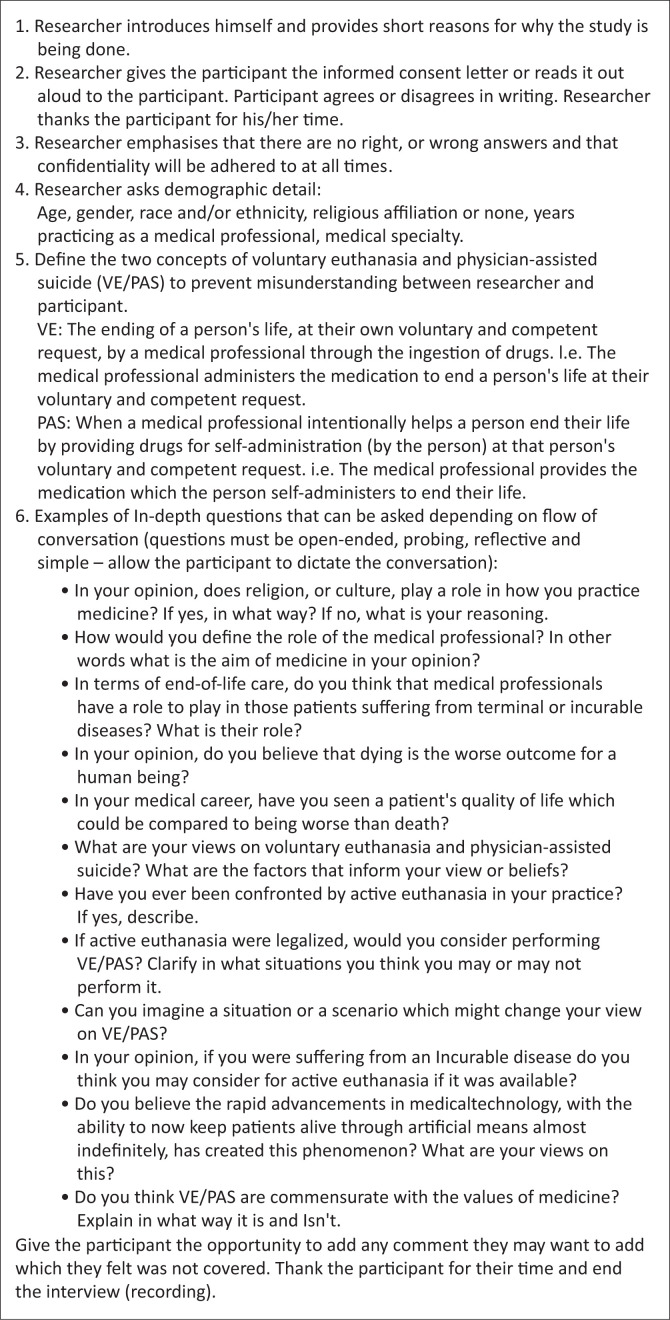
Guide to the in-depth interview used.

Semi-structured in-depth interviews were conducted to collect the data as in-depth interviews provide greater depth of a participants’ perceptions than, for example, a survey/questionnaire.^19^ Semi-structured interviews further allow for the discussion to follow the train of thought of the participant. The first part of the interview consisted of gathering demographic information. The second part consisted of open-ended questions with the intermittent use of focused questions to probe deeper into a participant’s views. The guide used to conduct the interviews is provided in Figure 1. Each interview was conducted in English and in a manner to protect the participants’ identity. Field notes (both descriptive notes and reflective notes) were made by the researcher following each interview. An audio recording of each interview (with the consent of the participant) was made using either software, if done online, or using a recording device if conducted in person. All interviews were transcribed verbatim, in English, by a third party who was remunerated by the researchers. The participants were anonymous to the third party.

### Data analysis

Audio recordings, field notes and verbatim transcriptions constituted the raw data. This was filed electronically and labelled appropriately using anonymous designations on the lead researcher’s password-protected home desktop. Informed consent forms (hardcopies) were kept in a file in a locked safe at the researcher’s home. Each interview was taken as a dataset on its own. The data were inductively analysed using a thematic analysis framework. After familiarisation, coding was conducted by the researchers for each dataset – where pertinent or recurring phrases or ideas were identified by coloured highlighting and then provided a code name. Each interview was also summarised. Following this, each interview was taken, and codes were grouped together to identify patterns. As the data-collection continued, it became clear that many of the patterns recurred or formed themes. Once saturation was achieved, all the identified themes from each individual interview were grouped, and general themes were formulated. These general themes were then reviewed, revised, finalised and interpreted.

Lincoln and Guba’s Evaluative Criteria were used to ensure the trustworthiness of the research.^20^ This involved establishing:

- Credibility – confidence in the truth of the findings.
- Transferability – showing that the findings have applicability in other contexts.
- Dependability – showing that the findings are consistent and can be repeated.
- Confirmability – a degree of neutrality or the extent to which the findings of a study are shaped by the respondents and not researcher bias, motivation or interest.

The findings of the research were credible as the transcriptions of the audio recordings were verbatim. Participants were given the opportunity to withdraw from the study at any time during or after the interview was conducted – if they felt the interview was not a credible representation of their views or experiences. Credibility was further ensured as once saturation was believed to be reached, additional participants were interviewed.

The findings were transferable as a diverse group of participants were purposively selected to ensure a good representation of South Africa’s multicultural medical community.

The results were dependable as participants were suitably diverse across a variety of different strata and participants were interviewed until saturation was reached.

Confirmability was ensured by not injecting the interview with bias – the researcher remained neutral, asked open-ended questions and allowed the participants the freedom to express their views anonymously and free of any undue pressure. The researcher was the only person who conducted the interviews to ensure consistency and reliability.

### Sample size

Ten research participants were interviewed as the initial analysis sample. This was based on Guest et al.’s finding that 10–12 in-depth interviews generally provided around 97% saturation.^21^ Francis et al.’s principles were used for specifying data saturation: if, after 10–12 interviews, three additional interviews lead to no new themes emerging, then this would be considered the point of saturation.^22^ Based on this, the minimum sample size was 10+3 participants. Additional participants would then have been interviewed until saturation was achieved. Within the study, saturation was reached with 10+3 participants as no new themes emerged after 10 interviews. Five interviews were conducted using online electronic software and eight interviews were conducted in person.

### Ethical considerations

Ethical clearance to conduct this study was obtained from the University of Pretoria’s Research Ethics Committee, with reference number 356/2021. Participants were anonymous to all but the researcher. Data were stored on a secure password-protected device.

## Results

Thirteen participants took part in the study as no new themes emerged after 10+3 interviews. Interview length time was between 27 min 34 s (shortest) and 61 min 13 s (longest). The average interview length was 38 min 31 s. Pertinent demographic information sought were:

- Age.
- Gender (as participant identifies with).
- Race/Ethnicity (as participant identifies with).
- Religious affiliation (if any).
- Years practicing as a medical professional.
- Medical discipline (currently).

Table 1 denotes the demographic information of the participants with their identification label.

**TABLE 1:** Participant Identification.

Participant identification	Age (years)	Gender	Race/ethnicity	Religious affiliation	Years practicing	Medical discipline
RM	54	Male	White person (cuban)	Christian	32	Critical care
NM	28	Female	Indian person	Islam	5	Family medicine
MM	29	Female	African person	Christian	6	Family medicine
AG	38	Female	Indian person	Hindu	12	Anaesthesiology
CS	57	Male	White person	Christian	32	Family medicine
ES	34	Male	White person	Christian	10	Psychiatry
HG	35	Male	Indian person	Hindu	9	General surgery
LR	36	Female	White person	Christian	9	Critical care
LS	47	Female	African person	Christian	22	Paediatrics
CD	29	Male	African person	None	7	Emergency medicine
MO	40	Male	African person	Christian	14	Oncology
FK	59	Male	Indian person	Islam	> 30	Family medicine
NZ	34	Female	African person	Christian	11	Internal medicine

The first part of the inductive thematic analysis process was to become familiarised with each dataset and/or interview. This was accomplished by reading the field notes, listening to the audio recordings and reading the transcripts of each interview multiple times to get a feel of how the participant perceived the topic under discussion. Each interview was then taken as its own dataset and meaningful phrases or statements were highlighted in colour. These phrases were subsequently taken, and a formulated meaning was given to each highlighted phrase or sentence or paragraph (coding). This represented a significant idea or view that the participant appeared to hold (a pattern) and the meaning of it often recurred throughout the interview.

The next part in the process, done concurrently with the coding process, was the summarising of each interview. This was to condense the interview, making it easier for the researchers to identify patterns (together with the formulated meanings derived from the coding process).

After the coding process and the summarising, themes were formulated for each interview by looking at common patterns that emerged from both the summaries and the significant phrases or sentences identified during the coding process.

It was clear, after the coding process, summarising and theme development of each individual dataset that certain themes recurred regularly. There was disagreement between medical professionals over whether active euthanasia should be legalised or remain illegal in South Africa. This divergence meant that some themes recurred in those broadly for euthanasia and some themes recurred in those broadly against euthanasia. There were also themes common to both. To this end, it was important to analyse the themes, irrespective of the underlying position of the participants. In total, four general themes, with sub-themes, were identified that required further analysis and interpretation. Although they will be discussed separately, there is a close relationship between them so that none can be understood in isolation. These four themes, with their sub-themes are discussed further in the text.

### Theme 1: Modern medical practice and euthanasia

Four sub-themes were identified under the theme of modern medical practice and euthanasia.

#### Sub-theme 1.1: Religion and medical practice are poorly compatible

Although many participants noted that religion often shapes the moral character of a person and cannot be completely divorced from medical practice, there was a general view that the religion of the medical professional should have no impact on medical practice or care:

‘… [*Y*]ou know religion helps me to make moral decisions about how I show up at work as a professional.’ (MM)

‘If I had to treat a Muslim or a Christian or an Atheist, the clinical work that I do, my religion doesn’t have an impact on what I will be doing to the patients.’ (LS)

In turn, the idea that the religion of the patient should be respected was the general view of most of the participants:

‘So, try and treat people according to their belief system, rather than according to your own.’ (ES)

Some of the participants stated that the medical professionals’ religion might play some role in medical practice:

‘Yes, I believe so … because I am a Christian, it will play a role, yes. And so, there are some things that I would do or not do for instance: I won’t do abortions … yes.’ (CS)

#### Sub-theme 1.2: Respecting autonomy is paramount

Respecting the patient’s autonomy within the doctor–patient relationship was mentioned by almost all the participants when discussing the role of religion in medical practice:

‘The patient’s values and the patient’s choices are paramount.’ (MO)

‘… [*E*]very person has the right to autonomy to decide what happens to him, because of that worth that we have as a person.’ (RM)

This was understood as the medical professional, engaging from a position of authority, not imposing their religious or moral values onto the patient but always acting in their best interest. Within the ambit of active euthanasia, the 13 participants had diverging views. Those participants broadly against active euthanasia often cited that autonomy, in the case of euthanasia, was not relevant (or limited) because what the patient was asking for was unethical – a belief often based on other factors such as religion or medical values, and so forth:

‘… [*M*]edical professionals are just as human as the patient and we have no right, as fellow human beings, to take someone’s life voluntarily or involuntarily.’ (NM)

The argument from respecting autonomy is regarded as one of the strongest arguments for euthanasia and was cited by those participants that were largely in favour of active euthanasia:

‘Everyone has the right to live, but everyone has the right to die.’ (LR)

#### Sub-theme 1.3: Aim of medicine is broad and holistic

All the participants in the study alluded to medicine being more than merely a curative endeavour. Most of the participants, irrespective of their views on VE/PAS, viewed the relief of suffering and the promotion of well-being and/or quality of life as the primary aim of medicine:

‘Aim of medicine is to actually help the patient … should comprise the whole holistic approach of the patient.’ (HG)

‘Aim of medicine is to heal, aim of medicine is to help somebody by the definition of the World Health Organization. Physical, mental, spiritual, psychological, all of the aspects of health of a person or family or society.’ (FK)

#### Sub-theme 1.4: Commensuration of euthanasia and medical values

Participants had contrasting views on whether euthanasia was commensurate with the values of medicine. All those against euthanasia felt that it was not as euthanasia was seen as doing harm to the patient, which would violate their oath to first do no harm:

‘I think euthanasia is against that, because you are stopping a life and nothing in medicine that we do up to now is intended to stop a life.’ (CS)

Those participants generally in favour of euthanasia had different views regarding whether medical values and euthanasia are commensurate. Some agreed that it is not commensurate with traditional medical values as currently understood but that it should be changed to reflect the current moral landscape. Other participants believed that euthanasia is in line with the values of medicine as the values of medicine are to alleviate suffering and to do good unto patients:

‘What I mean is, that if you follow by the book these values of do no harm and not to do anything that may cause harm to the patient … it would be against those values.’ (RM)

‘Yes, to relief the suffering, to increase the persons quality of life and to increase the wellbeing of the persons and to provide dignity.’ (ES)

### Theme 2: The problem of suffering in end-of-life care

The significance of suffering at the end of life was a theme prevalent in the interviews. Almost all participants remarked that they viewed intractable suffering as worse than death:

‘I think that suffering is the worse outcome for a human being. I think that living with pain that you cannot express to another person is worse than dying …’ (AG)

‘I think the worst outcome is pain for a human-being. To be in pain that is not eased, that is not …’ (NZ)

All the participants could think of patients they felt were worse off than being dead. Furthermore, many participants, irrespective of their views on VE/PAS, said they had often felt relief when a patient passed away as they believed it was better for them than to be in a state of continued suffering. The view that suffering also extended towards others such as family was notable in the interviews where participants regularly remarked that a state of continued suffering by the patient was also experienced by the family – and that death of the patient was often a relief for them:

‘… [*B*]ut I think, some people, some patients in terminal illnesses are suffering so much and are experiencing so much pain that … you know the family will even tell you that he is probably at peace now or you feel that the patient is suffering to no end.’ (NM)

The fear of suffering was also the reason all the participants generally in favour of active euthanasia said they would opt for euthanasia for themselves if it was available:

‘I definitely will, and I’m not sure whether I am answering this question whether I am in the medical profession, because you have to think about the background …’ (CD)

#### Sub-theme 2.1: Family is important in end-of-life care

A common sub-theme was that the family plays a critical role in end-of-life care and decision-making. Many of the participants felt that family were integral to any discussion regarding active euthanasia and that it would be impossible, within an African context, to discuss it solely within the patient–physician dyad. Any discussion of active euthanasia would, according to several of the participants broadly in favour of euthanasia, only be possible through a family-orientated approach:

‘So, in that setting it becomes not a medical decision but more like a family decision.’ (OM)

‘… [*I*]t is not just the patient; it is the patient’s family members that are also involved in this process.’ (LR)

‘… [*F*]amily knows what this person is going through.’ (MM)

### Theme 3: Euthanasia is complex

The dominant themes to emerge from the interviews were the complexity and multifaceted nature of euthanasia. This theme was divided into numerous sub-themes to do it justice.

#### Sub-theme 3.1: Euthanasia – Against religion and nature

All the participants, except one, that were broadly against euthanasia, discussed their opposition to euthanasia as being either against religious tenets or against nature. Most of the participants broadly against euthanasia remarked that euthanasia is murder as it is against God’s directives:

‘So basically, it is my Christian belief, that is one. Like it says: “Do not kill.” So, providing euthanasia, it basically means I am moving against what I believe in, I am killing a patient.’ (NZ)

‘Yes, that one is of course coming from a religious perspective, but it is God that will decide, that is the thing that I believe … Life is given by God; it will be taken by God.’ (FK)

Conversely, many of the participants in favour of euthanasia, and who identified with a certain religious affiliation, did not believe that it went against their religious beliefs:

‘I don’t necessarily see that as a conflict, no. Yes, I don’t, again something I haven’t thought about a lot before, but yes, I don’t see … I’m sure that there are a lot of people that would see a conflict, but me personally no.’ (ES)

In terms of the argument against euthanasia from nature – one that many participants alluded to – active euthanasia is considered an unnatural death as it is categorised as death by an external source:

‘I wouldn’t, I think a person should die at the time when they are supposed to die.’ (LS)

‘I do not have the power to decide that this patient should die now. It is not up to me. It is up to nature …’ (NM)

#### Sub-theme 3.2: Euthanasia – Killing and letting die

Many of the participants voiced a distinction between killing and letting die, arguing that letting someone die such as in the case of withholding or withdrawing treatment (passive euthanasia) was morally acceptable but actively ending the life of the patient (killing) was morally wrong – equated with murder:

‘Yes, I do. I do. It is a consent at murder.’ (NZ)

‘We as human being we have no right to terminate, or in other words kill another human being. I believe that strongly, not as a religious person but as a human being I have no right to kill any other person intentionally.’ (FK)

For those generally in favour of euthanasia, this distinction was irrelevant. If euthanasia was, in effect, killing a patient, it was seen as morally acceptable because it was the patient’s voluntary choice and carried out with good intentions:

‘No, because the motive I think as long as your motive is a good intention … And with compassion, in other words you do this with compassion to end a patient’s suffering, as compared to murder when one has different motives, not with compassion.’ (LR)

#### Sub-theme 3.3: Regulations and abuse

The need for a regulatory framework, if active euthanasia was ever legalised in South Africa, was a common thread throughout the interviews. Although participants differed in how strict this regulatory framework had to be, all those broadly in favour of euthanasia believed that it had to be regulated at some level. Some of those participants not in favour of legalising euthanasia felt that if it was to be legalised that it would have to be very tightly regulated to prevent abuse. Although none of the participants offered to guess what such a framework might look like, most felt that it would have to be conducted within a multidisciplinary team and that an application for euthanasia would have to be made that followed a deliberation process:

‘… [*I*]t should be a regulated process in which several specialists in the different fields involved in the condition that the patient has, are directly involved in that decision and also where specialists in the legal matters and medical ethics are also involved.’ (RM)

‘If in a country like South Africa, I would say no. I don’t see how it will be tightly regulated in a country like this one.’ (LS)

#### Sub-theme 3.4: Case-specific

All the participants who were broadly in favour of euthanasia, including some against it, stated that if euthanasia was legal or decriminalised it should only be available to specific patients – terminally ill patients, the severely suffering, when palliative care measures had failed, and so forth:

‘In cases, in distinct cases in a multidisciplinary team, yes …’ (HG)

#### Sub-theme 3.5: Grey areas

All the participants broadly in favour of euthanasia shared that there were grey areas within euthanasia that they were not comfortable with. Most participants felt uncomfortable about allowing euthanasia in children and in mentally ill patients. The problem in these patients, per the participants, was the lack of capacity of these individuals to voluntarily consent to active euthanasia:

‘Especially when patients are seen in the paediatric environment because, it is difficult to say in that instance, because you are dealing with a minor in this instance.’ (CD)

‘… [*H*]ow sure are we that the cognitive function is still intact for him to say that I want to end my life?’ (AG)

#### Sub-theme 3.6: Treatment exhaustion

The view that treatment options would have to be exhausted before euthanasia could be discussed in patients was a common view. This was true in both those broadly in favour of euthanasia and those broadly against it:

‘… [*M*]aking sure that all the possible alternatives of management have been exhausted and that all the relevant specialties and specialists are involved in the care of the patient, before reaching the conclusion that this patient is now a terminal patient in which we cannot do anything from the curative point of view.’ (RM)

#### Sub-theme 3.7: Unknown future

An interesting perspective was the idea of certainty among patients that they would not change their minds if they opted for euthanasia. One participant said that euthanasia should be considered only if it was highly unlikely that the patient would change their mind:

‘They might be in a better place, 2 months from the time they wanted to take the decision to have euthanasia or physician assisted suicide.’ (OM)

### Palliative care in South Africa is poor

A common theme espoused by all the participants was the importance of palliative care in terminal illness as well as the belief that palliative care has been very poorly practiced/accessible in South Africa.

‘In South Africa I think we are poor with palliative care. It is poor, it is very poor. In private hospitals it is poor, in public, in the state it is also poor.’ (NZ)

‘Pain is not adequately managed, dignity is not obtained, a lot of those times in those palliative centres you’ll find the patient in a soiled linen, you know from … It’s horrible, there is very little dignity at the moment so there is a very, very big opening for more focus on palliative care …’ (LR)

## Discussion

### Modern medical practice and euthanasia

#### Religion and medical practice are poorly compatible

It is broadly accepted in South Africa that no medical professional is legally obliged to perform procedures they find unconscionable unless it is an emergency.^23^ The obligation, however, is to then refer the patient to another medical professional or institution. This is different from the medical professional forcing their religious/moral views onto the patient from a position of authority. There was broad consensus amongst participants that the latter is unethical, and that the religion of the medical professional should have no bearing on medical practice or care.

#### Respecting autonomy

Autonomy refers to an individual’s right to live and make choices that are free from both controlling interferences by others and limitations that prevent such choice, such as poor understanding.^24^ The argument from respecting autonomy is regarded as one of the strongest in favour of active euthanasia with the major critique being that such radical individualism will lead to grave social consequences – especially with regard to marginalised or vulnerable individuals.^8^ Ultimately it is not clear how if someone has a constitutionally enshrined right to life, right to dignity and the right to control over one’s body – with the possible ability to forego the right to life – at least in cases of withholding and withdrawing treatment – the right to die with dignity is not a right too.^25^ It is perhaps strange that although suicide is not a crime in South Africa – often accomplished by particularly undignified methods – voluntary assisted suicide is considered murder under the law. Irrespective of this apparent paradox, most of the participants not in favour of euthanasia put forth the fact that although the right to autonomy is valid, it does not trump other factors such as the risk of abuse, palliative care, violations of medical values, societal harm, and so forth.^26^

#### Aim of medicine is broad and holistic

Modern medicine has often been characterised as a purely curative endeavour where the primary aim has been to cure the sick – and if a cure was not available, to seek one.^27^ This narrow definition of the aims of medicine was not traditionally considered to characterise medicine, and in recent years, there has been a concerted effort away from such a purely biomedical approach towards a biopsychosocial (holistic) model of medicine.^28^ This holistic approach to the aims of medicine was clear among all the participants and suggests that this is a normative belief among modern medical practitioners in South Africa.

#### Commensuration of euthanasia and medical values

All those against euthanasia felt that it was not commensurate with the values of medicine – which they broadly defined by the principles of beneficence and non-maleficence.^24^ Conversely, those that were in favour of active euthanasia believed that allowing continued suffering was a form of harm in itself and thus against the values of medicine. These two diverging views were striking. It could be argued by those against euthanasia that suffering can be alleviated completely without ending the life of the patient through terminal sedation – where a person is placed in a state of decreased awareness or unconsciousness until their death.^29^ Studies comparing terminal sedation and euthanasia in the Netherlands have found that patients who opt for terminal sedation do so more for reasons of alleviating severe pain, whereas those that opt for euthanasia do so out of a concern for preserving dignity.^29^ This suggests that terminal sedation may not be the answer for all cases of terminally ill patients.

### The problem of suffering in end-of-life care

Suffering is not only pervasive and multifaceted but also highly subjective. This idea was clear from the interviews in which all participants felt that at some level, intractable suffering could be considered a fate worse than death. Logically, this would mean that, even with the best palliative care services available, there will be limits as to what can be achieved – as it is dependent on the individual and the context. Even if it can be argued that the number of patients that cannot be palliated is few, it may be that: ‘reasonable people can, and do, sometimes refuse even high-quality palliative care’.^30^

#### Family in end-of-life care

Within the African context, the philosophy of ubuntu – a community-centric ethos – plays a central role within broader South African society and states that a sense of self can only be shaped by its relationship with other people.^31^ Family is thus a central part of the dying process, especially within the South African context. This idea, that dying is not a journey to be undertaken alone, was shared by the participants.

### Euthanasia is complex

#### Euthanasia – Against religion and nature

Traditionally, euthanasia would be against the prescriptions of most, if not all, mainstream religious tenets as euthanasia would be seen as murder – against the authority of a deity or ‘God’. The idea that assisted suicide is akin to ‘playing God’ is not new and has always been an argument brought by those arguing against euthanasia.^8^ Religion has also been shown, in the literature, to be the primary factor in those opposing euthanasia.^11^ The crux of the argument from religion is that human life is sacred: it is given by ‘God’ and can thus only be ended by ‘God’. This belief was pervasive in those participants against euthanasia. Regardless of this, the question is whether religion should be considered in any discussion of euthanasia, as South Africa is a secular state with a variety of diverse religions that are constitutionally protected.^32^ One person’s religious beliefs thus cannot trump another’s religious beliefs or lack thereof.

In terms of active euthanasia being against nature, for many participants against euthanasia, there was an important difference between a natural and an unnatural death – where active euthanasia is viewed as an unnatural death and thus morally wrong.^33^ In contrast, passive euthanasia (withholding and withdrawing treatment) is seen as a form of natural death, and thus not morally wrong, as no external force causes the death: it is simply the removal of life-sustaining measures.^33^

#### Euthanasia – Killing and letting die

Instinctively, it feels as if a moral distinction between killing someone and letting someone die exists.^34^ Although mentioned by participants, this argument rests on the assumption that killing a person is morally worse than letting a person die. James Rachels, in a seminal paper, argued that, all things considered equal, there is no moral distinction between killing and letting die as cases exist in which letting die may be considered morally worse than killing.^35^ Winston Nesbitt argues that what should be considered is the ‘willingness to kill’ or the intention with which such killing or letting die is conducted.^34^ It is this concept of intention that divided the participants on euthanasia as those in favour of euthanasia believed that it is morally acceptable because of the good intentions with which it will be performed.

#### Regulations and abuse

If active euthanasia was legalised, it would have to be regulated at the level of the state in South Africa through a suitable regulatory body. This belief was common among participants both in favour and against legalising euthanasia. The fear was the risk of abuse, an idea espoused in the literature too.^4,8^ In their opposition to the case brought by Stransham-Ford, the state argued that legalising active euthanasia would lead to a slippery slope of abuse.^4^ This would imply that the state itself does not have confidence in their ability to regulate and police active euthanasia in South Africa.

#### Case-specific

Although there is disagreement regarding which cases might hypothetically be considered for euthanasia and which will not among participants, there was a sense that a thorough and prolonged deliberation process would be required to objectively conclude whether someone – who has made an application for active euthanasia – may proceed with the procedure. This deliberation process is similar to what already occurs in countries where euthanasia is legal.^36^

#### Grey areas

There was general unease among participants when discussing active euthanasia within the paediatric and mentally ill population. Globally, it is also controversial.^1,37^ In the Netherlands, where it is legal to perform active euthanasia in children and patients with mental illness, a thorough process must be followed and applicants must have a diagnosed medical condition – being tired of life cannot be accepted as a reason for euthanasia.^38^ A very recent case in the Netherlands, of Lauren Hoeve, a 28-year-old diagnosed with Myalgic Encephalomyelitis, who was successfully euthanised on 27 January 2024, has reignited the controversy.^39^

#### Treatment exhaustion

Many of the participants broadly in favour of euthanasia, who felt that treatment options would have to be exhausted before euthanasia could be considered, worked in the public sector. Resources in this sector are highly limited in South Africa and palliative care is not well funded or resourced – the public sector is responsible for treating the overwhelming majority of the population.^40^ It is not clear how many, if any, patients in the public sector would ever be able to access euthanasia if the requirement was that all treatment options, including palliative care options, have been exhausted.

#### Unknown future

The future – in the scenario of a patient requesting euthanasia – is unknowable and cannot be known with certainty until the future becomes the present (by which time it is not the future any longer). It is a philosophical question that arguably has little significance in practice for we can only know what we know based on the past and present and cannot reasonably base decisions of such magnitude on how we may potentially feel in the future – otherwise we would stagnate and never make decisions of any import.

### Palliative care in South Africa is poor

It is estimated that 78% of the global population in need of palliative care reside in middle- and low-income countries and that only 14% of the global population has access to proper palliative care services and resources.^41^ It is thus not surprising that palliative care in South Africa is poor – a fact mentioned by all participants. This is especially true as the majority of palliative care services in the country are non-governmental organisation (NGO) driven – studies have shown that only 3% of all palliative care organisations in South Africa can be found in the public sector.^40^ The view that euthanasia cannot be implemented in South Africa as palliative care is too poor was common. The concern was that euthanasia would be abused or would be chosen by people only because they do not have access to proper resources to alleviate their suffering. This concern is, perhaps, a valid argument against legalising euthanasia in South Africa. Conversely, it could be argued that by not providing people with adequate palliative care and by not providing them with the ability to voluntarily be assisted in dying, a person is ultimately left worse off. Is it fair for society to deny someone the ability to be assisted in dying if palliative measures have failed – as not all have equal access to the same resources – when the situation of poor access to palliative care is the result of the very same society not investing in adequate palliative care?

### Limitations of study

The results of the study were dependent on the experience of the researcher in conducting interviews and extracting valuable information from the vast data that were generated. Regarding reflexivity, the researcher acknowledges their own biases that, although actively ameliorated through asking open-ended questions, may still have influenced the results as it may have influenced participants to answer in certain ways. Purposive sampling was used instead of random sampling. Although this was used to ensure rich data, this could impact the generalisability of the findings. The lack of participants identifying as mixed race or Asian South Africans in the study was also a limitation.

### Recommendations

Despite saturation being reached in this study, it is possible that larger qualitative studies with a greater number of participants, especially including more palliative care physicians and oncologists, may provide an even greater understanding of these themes or perhaps new ones. Newer quantitative studies, exploring the views of medical professionals towards euthanasia will be able to study whether there are more favourable views amongst medical professionals now (as appears to be the case with medical undergraduates) versus more than a decade ago. Given South Africa’s cultural and religious diversity, stratified studies comparing different demographic groups may also yield valuable information.

## Conclusion

Medical professionals in South Africa have wide-ranging views regarding active euthanasia. Different medical professionals place different emphases on certain parts of the active euthanasia debate. Religion was a major issue in those that objected to euthanasia, as well as the belief that it ran contrary to medical values as it violates the duty to do no harm. In those that were generally in favour of euthanasia, the overriding belief was that medical professionals have a primary duty to alleviate suffering – even if it means ending the life of the patient out of good intentions – and that to not assist someone in dying, who is voluntarily requesting it, might constitute a harm in and of itself.

The significant problem of suffering in end-of-life, as well as the importance of family, was a common thread among the participants. There was also a common consensus that palliative care in South Africa is very poor and that this is integral to any discussion regarding active euthanasia.

The future of active euthanasia in medical, ethical, social, legal and political discourse in South Africa is uncertain. Given the rapidity with which nation states across the globe are legalising the practice, and the increasing number of legal cases ongoing in South Africa, this is not a debate that is likely to disappear. Improved understanding of the complexity of the issue within the 21^st^-century South African context – given the current dearth of contextual research on the topic – is imperative to ensure rational and sensible decision-making moving forward as it involves some of the most vulnerable people in society, the terminally ill.
